# Phase of Topological Lattice with Leaky Guided Mode Resonance

**DOI:** 10.3390/nano13243152

**Published:** 2023-12-16

**Authors:** Heejin Choi, Seonyeong Kim, Markus Scherrer, Kirsten Moselund, Chang-Won Lee

**Affiliations:** 1 Institute of Advanced Optics and Photonics, Hanbat National University, Daejeon 34158, Republic of Korea; 28802chj@hanbat.ac.kr; 2 Department of Physics, Sejong University, Seoul 05006, Republic of Korea; 3 Laboratory of Nano and Quantum Technologies, Paul Scherrer Institut, 5232 Villigen, Switzerland; seonyeong.kim@psi.ch (S.K.); kirsten.moselund@psi.ch (K.M.); 4 Institute of Electrical and Micro Engineering, École Polytechnique Fédérale de Lausanne, 1015 Lausanne, Switzerland; 5 IBM Research Europe—Zürich, 8803 Rüschlikon, Switzerland; mas@zurich.ibm.com; 6 Department of Applied Optics, School of Basic Sciences, Hanbat National University, Daejeon 34158, Republic of Korea

**Keywords:** topological invariants, Zak phases, non-Hermitian, guided-mode resonance, photonic crystal

## Abstract

Topological nature in different areas of physics and electronics has often been characterized and controlled through topological invariants depending on the global properties of the material. The validity of bulk–edge correspondence and symmetry-related topological invariants has been extended to non-Hermitian systems. Correspondingly, the value of geometric phases, such as the Pancharatnam–Berry or Zak phases, under the adiabatic quantum deformation process in the presence of non-Hermitian conditions, are now of significant interest. Here, we explicitly calculate the Zak phases of one-dimensional topological nanobeams that sustain guided-mode resonances, which lead to energy leakage to a continuum state. The retrieved Zak phases show as zero for trivial and as π for nontrivial photonic crystals, respectively, which ensures bulk–edge correspondence is still valid for certain non-Hermitian conditions.

## 1. Introduction

Topological photonics, through the means of topological invariants and bulk–edge correspondence, allows for the manipulation of light in a robust way that is immune to disorders [1,2]. These topological invariants, a cornerstone in condensed matter physics, not only describe the properties of the bulk material but also predict the presence of localized states at interfaces between different bulk materials. The phenomenon of localized states, referring to the bulk–edge correspondence, confines light within a specific region [3,4], effectively creating a nonconventional cavity [5,6,7]. This confinement enables the light to serve as a beam emitter [8], similar to the operation of lasers [9,10,11]. By implementing such photonic structures, analogous to electronic systems, photonic topological key functionalities can be achieved despite the presence of non-Hermitian characteristics, including leakage [12,13,14].

The significance underlying the bulk topological invariant in Hermitian systems is expounded through the concept of the bulk–edge correspondence, where the bulk governs the topological properties of the edge modes. This correspondence establishes a nexus between the topological attributes prevalent within the bulk material and the inherent properties of its boundaries. This leads to amalgamating materials characterized by distinct topological invariants and an energy gap that necessitates closure within the interface, thereby facilitating the emergence of localized states. So far, a number of topological photonic structures with localized edge states have been realized by the attachment of two lattices with different topological invariants, like the Jackiw–Rebbi state [9,15,16,17]. This state engenders pronounced lateral confinement and delivers distinct spectral signatures.

In non-Hermitian photonic systems, leakage assumes a significant role, acting as a conduit for interaction with the external environment. This leakage phenomenon presents a fascinating opportunity where it can serve as a window through which highly refined light can be emitted from within a structured material. Even though leakage might decrease the validity of the bulk–edge correspondence, the light emitted by the leak can be harnessed to characterize the material or facilitate experimental measurement of topological invariants in the far-field [18,19,20]. To achieve this, far-field spectra are observed for topological invariants, or bulk-edge correspondences are experimentally established.

Remarkably, the validity of topological invariants extends to photonic platforms involving leaky non-Hermitian systems, such as plasmonics [21,22,23,24], waveguide arrays [25,26], and photonic crystals (PhCs) [27,28,29,30]. Among these platforms, PhC slabs have gained significant popularity for exploring topological phenomena of light, particularly guided⁠-mode resonances (GMRs) [31]. GMRs are characterized by their electromagnetic power being tightly confined within the slab while also being capable of coupling with external radiation, leading to leaky modes that can interact with extended states and radiate light [8,32].

In this study, we calculate Zak phases directly from the mode profiles at the band edge corresponding to the GMR frequency in the PhC slab structure. To achieve this, we used the simple one-dimensional (1D) Su–Schrieffer–Heeger (SSH) [33] model, which has been recognized as a topological prototype. The Hermitian SSH model yields topological invariants quantized to values of 0 and π through the Zak phase [34], and leaky SSH structures show varying Zak phases, which implies that the geometric phase might be used to characterize or categorize the non-Hermitian conditions [23]. We calculate the Bloch modes of the magnetic field (*H_z_*) obtained from the finite-difference time-domain (FDTD) method in the first Brillouin zone (BZ) and use them to obtain Zak phases of the lowest band for trivial and nontrivial photonic structures [35,36].

## 2. Methods

**Figure 1:**
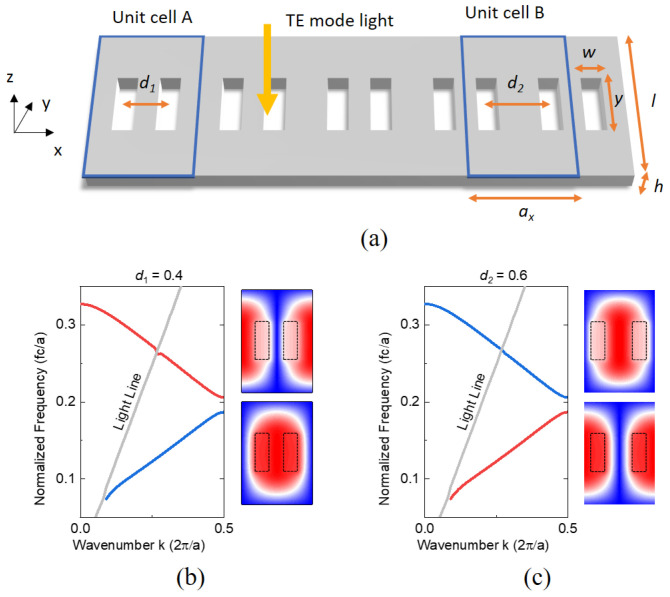
(**a**) Schematic of 1D leaky PhC structure implementing the SSH model. The 1D SSH model is composed of dimerized holes in a slab structure. The dimerized holes in the unit cell are arranged by the normalized distance *d*_1_ (unit cell A) and *d*_2_ (unit cell B). Two possible unit cells were chosen such that the band inversion occurs when the sum of distances *d*_1_ and *d*_2_ is one. (**b**,**c**) The photonic band structures and magnetic field (*H_z_*) mode profiles for PhCs composed of unit cells A with *d*_1_ = 0.4 (**b**) and B with *d*_2_ = 0.6 (**c**), respectively. The red and blue colors of the band curve indicate the inversion. The upper and lower right figures show the magnetic field (*H_z_*) Bloch mode profile of the upper and lower band edge. The band structures for the unit cells A and B are identical; however, band inversion occurs. The spatial magnetic field (*H_z_*) distributions of the band edge at *k* = 0.5 are illustrated. The grey line corresponds to the light line. The structure is defined by various parameters: lattice constant *a_x_* = 270 nm, hole width *w* = 54 nm, hole length *y* = 162 nm, slab width *l* = 432 nm, slab height *h* = 172.8 nm, slab index *n_slab_* = 3.4.

A one-dimensional PhC slab is composed of air holes and a high-refractive index material, as shown in Figure 1a. The photonic version of the SSH model can be made of dimerized holes with a normalized distance *d* by *a_x_*. The intracell and intercell hopping are represented as *d*_1_ (unit cell A) and *d*_2_ (unit cell B), respectively. Due to the system’s inversion symmetry, the Zak phase under Hermitian conditions for each distinct energy band has a quantized value. However, the periodically modulated refraction leads to GMRs, which can couple to external leaky radiation.

The eigenfunctions of holey PhC structures are TE modes, allowing us to use a z-directional magnetic field (*H_z_*) to calculate Zak phases. In the absence of external currents and sources, Maxwell’s equations can be used to solve the following equation [37]:

(1) $$\nabla \times \left[\frac{1}{\varepsilon \left(r\right)}\nabla \times H_{z}(r)\right]={\left(\frac{\omega }{c}\right)}^{2}H_{z}(r),$$

For TE mode, Equation (1) is expressed as an eigenvalue problem in terms of the magnetic field,$H_{z}(r)$. Owing to the periodicity of PhC, the solutions of Equation (1) can be expressed using the Bloch theorem.

(2) $$H_{z,k}\left(r\right)=e^{ik\cdot r}u_{k}\left(r\right)=e^{ik\cdot r}u_{k}(r+a)$$

where $u_{k}(r)$ is the Bloch function of the lattice with the lattice periodicity **a**.

With Ansys-Lumerical FDTD solutions with a TE source launched into the PhCs, we calculated the band structures for unit cells A and B. The structures of the lowest and the first excited bands, as well as the corresponding Bloch modes, are drawn in Figure 1b and 1c, respectively. The emergence of grey light lines from the slab structure indicates the presence of leaky modes. The upper and lower right figures illustrate the Bloch mode of the magnetic field (*H_z_*) at each band edge, respectively. Although the band structures of the two PhCs are identical, the Bloch mode profile of the magnetic field (*H_z_*) at the band edge suggests band inversion between the two PhCs. This inversion occurs when the sum of distances d between the two holes of unit cells A and B equals unity.

Even though the band structure of infinite PhCs differs from that of finite structures, the resonance spectrum can still be observed based on GMRs in a finite structure consisting of a certain number of unit cells. It becomes crucial to determine the number of unit cells that yield identical band gaps between finite and infinite structures. We find that when there are more than 40 unit cells, the band gap at the band edge calculated in the infinite structure aligns with the band gap in the transmission spectrum of the finite structure. This allows a direct comparison of the topological natures of an infinite structure from a sufficiently long finite structure with a leaky mode.

**Figure 2:**
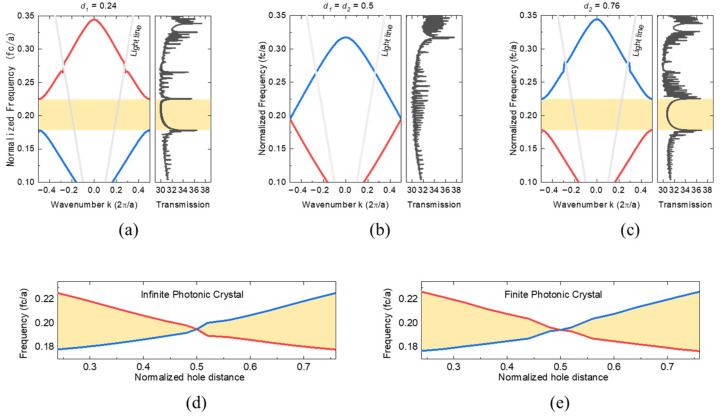
(**a**–**c**) Photonic band structure and transmission spectra for three conditions: *d*_1_ = 0.24, *d*_1_ *= d*_2_ = 0.5, and *d*_2_ = 0.76. The band gap at the band edge, which is obtained from PhCs with an infinite structure (**left**), is identical to the one from the transmission spectrum of a finite structure consisting of 40 unit cells (**right**). The yellow regions are photonic band gaps. (**d**,**e**) The span of the band gap as a function of the normalized hole distance *d*. For both infinite (**left**) and finite (**right**) PhC structures, the yellow region illustrating the band gap size becomes smaller as the normalized hole distance closes to 0.5. After band crossing occurs at *d* = 0.5, the band gap size increases gradually, showing the band inversion. The red and blue colors of the band curves indicate the inversion.

Figure 2 displays the calculated photonic band structures and transmission spectra for different values: *d*_1_ = 0.24, *d*_1_ *= d*_2_ = 0.5, and *d*_2_ = 0.76. The band gap at the band edge obtained from PhCs with an infinite structure (left) matches precisely with the transmission spectrum of the finite structure (right). For the transmission spectra, a monitor was placed on the surface of a nanobeam structure consisting of 40 unit cells, corresponding to the middle 20 unit cell area. A TE mode dipole source was then launched onto the nanobeam to find a resonance peak in the 150~500 THz range. These bands meet at the band edge at *d*_1_ *= d*_2_ = 0.5, resulting in a Dirac⁠-like point formation and band crossing.

To observe the evolution of the photonic band gap, we gradually span the value of *d*_1_ and *d*_2_ for both infinite and finite PhCs, as illustrated in Figure 2d,e. For *d*_1_, we can observe an open band gap in both infinite and finite PhCs, and vice versa for *d*_2_. However, at *d* = 0.5, the photonic band gap closes, indicating the appearance of a Dirac⁠-like point. The extension of the yellow region in the plot indicates that the photonic band is proportionally related to the differences in distances.

## 3. Results

The Zak phase is given as an integral over the BZ:

(3) $$\phi_{n}=i\oint \left\langle u_{n,k}|\nabla_{k}|u_{n,k}\right\rangle \cdot dk$$

where *n* is the band index. In a continuum limit, the analytical form of the Bloch functions for each *n* is required to be known. However, finding the Zak phases for arbitrary photonic structures requires some manner of discretization. If we discretize the BZ into N intervals, the Zak phase for each band can be recast in the following form [38]

(4) $$\phi =-Im\left[\ln\left[\left\langle u_{k_{1}}(r)|u_{k_{2}}(r)\right\rangle \left\langle u_{k_{2}}(r)|u_{k_{3}}(r)\right\rangle \cdots \left\langle u_{k_{N-1}}(r)|u_{k_{N}}(r)\right\rangle \right]\right]$$

Here, we note that can be extracted from Equation (2) by changing the phases:

(5) $$u_{k}\left(r\right)=e^{-ik\cdot r}H_{z,k}\left(r\right)$$

where the magnetic field profiles for each spatial and wave number location can be obtained from FDTD simulation. In principle, the intervals between the adjacent wave numbers do not need to be equidistant to calculate the entire bra–ket products. However, the relative phases for bra–ket values should be compensated for by Equation (5) accordingly. Therefore, we used the simplest subroutine with equal intervals of wave numbers in the FDTD simulator.

**Figure 3:**
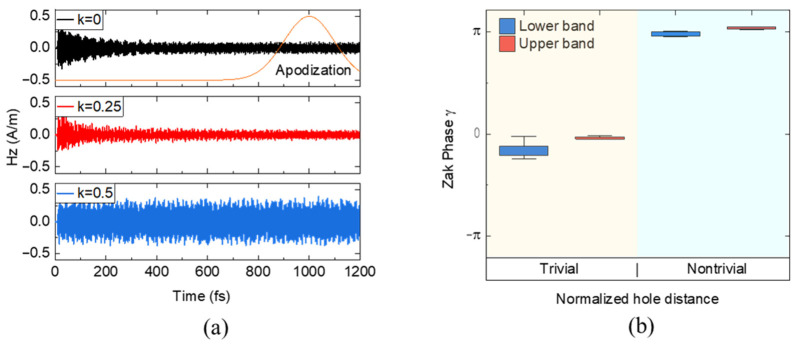
(**a**) Time evolution of the magnetic field (*H_z_*) leaky mode in the PhCs for *k* = 0.5 (blue), *k* = 0.25 (red), and *k* = 0 (black). After the decaying magnetic fields reached a uniform state at *k* = 0 and 0.25, the *H_z_* mode profile was obtained at 1000 fs. At *k* = 0.5, the magnetic field survives without decay, which indicates the resonance behavior. (**b**) Calculated Zak phase *γ* of each band for the cases of *d* = 0.4 (trivial) and *d* = 0.6 (nontrivial).

Unlike the finite element method, a pulse-like excitation source is required to obtain the Bloch modes in the FDTD simulation. Hermitian Zak phase assumes a steady state after equilibrium is established. Therefore, it is crucial to examine how fast the electromagnetic fields decay in our structure to retrieve Zak phases. To observe the decay characteristics of different electromagnetic modes in our PhC, we represent the temporal evolution as spatial averages in the unit cell, as depicted in Figure 3a, after which the monitored magnetic field gradually diminishes. After the dipole source generated a pulse during 0 < *t* < 4.98 fs, the monitored magnetic field decays sufficiently for every wave number value. Once the varying magnetic fields attain a uniform state, the Bloch mode profile was obtained at 1000 fs with an apodization time width of 250 fs. To avoid any influence from the light source, we apply a Gaussian function with a Full-Width Half-Maximum (FWHM) of 250 fs as an apodization window. This step facilitates the exclusion of light source effects from the analysis.

For small wave numbers such as *k* = 0, the spatial average decays quickly, and the steady state is already established right before 400 fs, as shown in the top figure of Figure 3a. For *k* = 0.25, the field also decays similarly. However, for large wave numbers such as *k* = 0.5, where the band edge is, the GMRs ensure the persistence of the field amplitude enough to prolong it for more than 1200 fs. This means the GMRs support a strongly confined mode in the PhCs plane. When an edge or lattice distortion exists, this GMR mode can be leaked out from the planar PhC to the far⁠-field.

To numerically compute the Zak phase, we extracted the Bloch functions for *d* = 0.4 and *d* = 0.6, which were then inserted into Equation (4). As shown in Figure 1, there is no degeneracy in the lower and upper bands, allowing us to confidently apply Equation (4) for our calculations. With the data of the *H_z_* field obtained at discrete points in the BZ, we defined the first BZ and discretized it into a regular mesh in k-space. Specifically, we discretized the BZ into N = 100 equal intervals for the *x*-axis and M = 160 for the *y*-axis. Subsequently, we computed the inner products with the adjacent values from $|\left.u_{{n,k}_{-0.5}}\right\rangle$ to $|\left.u_{{n,k}_{0.5}}\right\rangle$. This process generated a distribution of Zak phase values along the *y*-axis, effectively summarizing the results of multiple calculations.

The resulting Zak phase distribution along the hole length *y* for the first BZ is illustrated in Figure 3b. Specifically, for PhCs with *d*_1_ = 0.4 and *d*_2_ = 0.6, the Zak phase values are almost quantized to 0 and π, respectively, even with GMR leakage. We find that the finite size effect along the hole length in the *y* direction shows some distribution of the Zak phases. We represent this distribution as the y-scale error bar in Figure 3b. Calculating the Zak phase for the lower band of each PhC proves to be more challenging due to the merging of the lower band and light line near *k* = 0, which leads to larger error bars. For PhCs with *d*_1_ = 0.4, both the lower and upper bands exhibit a Zak phase close to 0, indicating trivial behavior. Conversely, for PhCs with *d*_2_ = 0.6, the Zak phase takes on a value of π, signifying nontrivial behavior. In the GMR structure, the Zak phase relies directly on the mode profiles of the magnetic field, enabling a straightforward approach to determine the topological invariants. The most intriguing aspect of this result is that, despite being non-Hermitian, the topological geometric phases still show good quantized values, unlike the previously reported non-Hermitian calculation [19].

**Figure 4:**
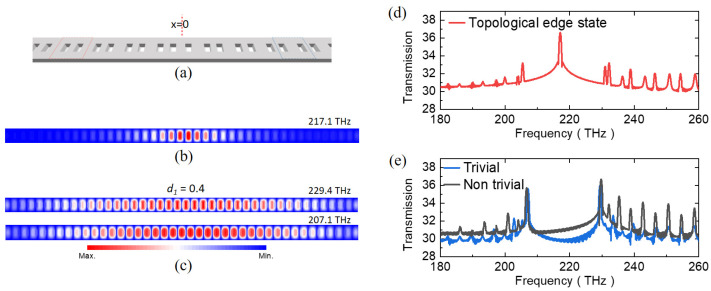
(**a**) The schematic representation of a photonic junction structure comprising both trivial and nontrivial PhCs, facilitating a bulk–edge correspondence. (**b**) The mode profile of the magnetic field (*H_z_*) encompasses the entire structure and is indicative of the junction configuration. Notably, a distinctive topological edge mode becomes confined to the interface between two distinct PhCs, manifesting at a frequency of 217.1 THz. (**c**) The magnetic field (*H_z_*) mode profiles at the upper and lower states of the spectral band. (**d**,**e**) The transmission spectra of the photonic junction structure correspond with those of finite structures possessing either trivial or nontrivial phases, respectively. Of significance, the transmission spectrum exhibits a prominent peak aligned with the topological attributes, further affirming the concurrence between bulk and edge characteristics. This topological spectral peak emerges precisely at 217.1 THz, coinciding with the central point of the band gap, which spans the frequency range of 207.1 to 229.4 THz.

When attaching two PhCs characterized by a trivial Zak phase of 0 and a nontrivial Zak phase of π, as shown in Figure 4a, localized modes centered at the edge emerge, as shown in Figure 4b,c, depending on the optical frequency. Corresponding transmission spectra showing a topological edge mode clearly emerge, as shown in Figure 4d,e. For structures characterized as either entirely trivial or nontrivial, the computed magnetic field distribution at each band edge at 207.1 and 229.4 THz exhibits propagation towards the exterior. On the other hand, exactly at the peak frequency of 217.1 THz, a more confined mode emerges, spanning just a few unit cells.

**Figure 5:**
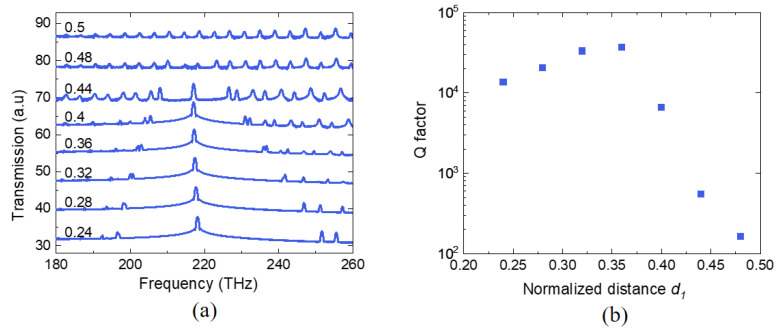
(**a**) Spectral peaks at the topological edge state, which are generated at the interface with varying distances *d*_1_. The Jackiw⁠–Rebbi state spectral peak retains the same frequency of 217.1 THz, regardless of the values of *d*_1_. (**b**) Calculated Q factors (blue squares) of the edge modes for different *d*_1_ values. The maximum value of the Q factor is 37,000 at *d*_1_ = 0.38.

The resilience of the topological edge state occurring at the boundary between two PhCs possessing distinct Zak phases can be checked by varying *d*s. Due to the presence of inversion symmetry, the spectral peak of the topological edge state remains consistently positioned at the same frequency, regardless of the values of *d*_1_, as shown in Figure 5a. This demonstrates that the system maintains its inversion symmetry, thereby ensuring a high degree of stability in the frequency of the edge mode. This implies that the eigenfrequency of the edge state remains unaltered as long as the integrity of the bulk Zak phase is upheld.

On the other hand, the Q-factor, an indicator of energy leakage through GMR, is strongly influenced by *d*_1_ (and *d*_2_), which is shown in Figure 5b. The Q-factor increases as a function of *d*_1_ up to 0.38, with the highest value being 38,000, then decreases rapidly. This is because the mode size becomes larger compared to the simulation domain, with increasing *d*_1_. This leads to additional leakage to the boundary of the simulation domain. We note that the rapid decay of the Q factor for specific *d*_1_ values (like 0.36) aligns with the previous findings on topological nanocavity lasers [10]. Eventually, the Q-factor as a function of *d*_1_ should increase and be saturated up to a maximal value in the presence of leakage. However, with the finite number of unit cells in the simulation domain, we could not observe the increasing feature of the Q-factor with our limited simulation time.

## 4. Conclusions

In summary, we numerically calculated Zak phases of 1D nanobeams with leaky guided-mode resonances by discretizing Block magnetic fields in the first BZ. The quantized values of 0 or π were obtained for trivial and nontrivial PhCs, respectively. The presence of coexisting leaky modes was observed in the band structure, leading to a resonance spectrum in line with the implementation of the SSH model. The topological Zak phases extracted from our model non-Hermitian system were consistent with those derived from the Hermitian counterpart.

Furthermore, we showed the presence and resilience of bulk⁠–edge correspondence, a phenomenon achieved by establishing a link between bulk materials possessing distinct topological invariants. This was tested by the identification of a topological edge state spectral peak at the junction of the PhC slab, where trivial and nontrivial phases intersect. The resonant characteristics of this topological edge mode show consistent optical frequency, regardless of the dimerized distances in the SSH PhCs. Our calculation may offer insights regarding engineering interface states made of two types of PhCs by investigating global topological properties in the presence of energy leakage leading to finite Q-factor.

## Data Availability

The data presented in this study are available upon request from the corresponding author.
